# Impacts of Eccentric Resistance Exercise on DNA Methylation of Candidate Genes for Inflammatory Cytokines in Skeletal Muscle and Leukocytes of Healthy Males

**DOI:** 10.3390/genes14020478

**Published:** 2023-02-13

**Authors:** David John Hunter, Lynsey S. James, Bethan Hussey, Richard A. Ferguson, Martin R. Lindley, Sarabjit S. Mastana

**Affiliations:** 1 Translational Chemical Biology Research Group, School of Sport, Exercise and Health Sciences, Loughborough University, Epinal Way, Loughborough LE11 3TU, UK; 2 National Centre for Sport and Exercise Medicine, School of Sport, Exercise and Health Sciences, Loughborough University, Epinal Way, Loughborough LE11 3TU, UK; 3 School of Biomedical Sciences, University of Birmingham, Edgbaston, Birmingham B15 2TT, UK

**Keywords:** DNA methylation, epigenetics, resistance exercise, fatty acids, skeletal muscle, leukocytes, inflammation, *IL6*, *TNF*

## Abstract

Physical inactivity and a poor diet increase systemic inflammation, while chronic inflammation can be reduced through exercise and nutritional interventions. The mechanisms underlying the impacts of lifestyle interventions on inflammation remain to be fully explained; however, epigenetic modifications may be critical. The purpose of our study was to investigate the impacts of eccentric resistance exercise and fatty acid supplementation on DNA methylation and mRNA expression of *TNF* and *IL6* in skeletal muscle and leukocytes. Eight non-resistance exercise-trained males completed three bouts of isokinetic eccentric contractions of the knee extensors. The first bout occurred at baseline, the second occurred following a three-week supplementation of either omega-3 polyunsaturated fatty acid or extra virgin olive oil and the final bout occurred after eight-weeks of eccentric resistance training and supplementation. Acute exercise decreased skeletal muscle *TNF* DNA methylation by 5% (*p* = 0.031), whereas *IL6* DNA methylation increased by 3% (*p* = 0.01). Leukocyte DNA methylation was unchanged following exercise (*p* > 0.05); however, three hours post-exercise the *TNF* DNA methylation decreased by 2% (*p* = 0.004). In skeletal muscle, increased *TNF* and *IL6* mRNA expression levels were identified immediately post-exercise (*p* < 0.027); however, the leukocyte mRNA expression was unchanged. Associations between DNA methylation and markers of exercise performance, inflammation and muscle damage were identified (*p* < 0.05). Acute eccentric resistance exercise is sufficient to induce tissue-specific DNA methylation modifications to *TNF* and *IL6*; however, neither eccentric training nor supplementation was sufficient to further modify the DNA methylation.

## 1. Introduction

An acute inflammatory response, characterised by elevated levels of pro-inflammatory cytokines such as IL-6 and TNFα [1], is required to minimise the damage triggered by pathogens, damaged tissues and toxic compounds to maintain tissue homeostasis [2]. When the acute inflammatory response is uncontrolled it develops into chronic inflammation, which is a risk factor for several diseases including cancer [3,4], type-2-diabetes [5], cardiovascular disease [6] and myopathies [7,8,9]. Inflammation can also be influenced by environmental factors; a lack of physical activity and a poor diet increase the expression of pro-inflammatory cytokines [10], whereas exercise and nutritional interventions have anti-inflammatory properties and can be used as effective treatments for inflammatory disorders [1,11,12,13]. The molecular mechanisms responsible for the interactions between lifestyle factors and inflammation remain to be fully explained; however, the reversible environmental impact of physical activity indicates that epigenetic modifications may be critical in the regulation of inflammatory processes.

The DNA methylation status of pro-inflammatory cytokines is associated with various inflammatory diseases, including *TNF* with type 2 diabetes [14] and Alzheimer’s disease [15] and *IL6* with rheumatoid arthritis [16] and obesity [17]. Exercise training is associated with various health outcomes, including a reduction in chronic systemic inflammation [1,18,19]. Conversely, acute exercise, particularly in individuals who are unaccustomed to the stimulus, causes local damage to the working muscles, with greater damage occurring when eccentric contractions are performed [20,21,22]. The muscle damage response increases the expression of pro-inflammatory cytokines, including IL-6 and TNF-α [22,23], and stimulates leukocyte infiltration into the muscle, which further attracts macrophages to remove the damaged muscle fibres and leads to the release of various growth factors that regulate satellite cell proliferation differentiation [24]. Unlike chronic inflammation, which is associated with skeletal muscle atrophy via the hypermethylation of *MyoD* [25], the expression of cytokines following acute exercise is critical for the repair, regeneration and hypertrophy of skeletal muscle [9,26,27]. Resistance exercise is sufficient to modify the mRNA expression of *IL6* and *TNF* in skeletal muscle, but not leukocytes [28]. The tissue-specific transcriptional changes following resistance exercise suggest that epigenetic mechanisms may control the exercise-induced production of cytokines; however, there is a lack of studies investigating the DNA methylation of these critical cytokines in response to muscle-damaging exercise.

The majority of the literature investigating the impacts of exercise on DNA methylation has focused on the impacts of aerobic training [29,30,31,32,33,34] and acute bouts of aerobic exercise [35,36,37,38,39], whereas limited studies exist regarding the epigenetic consequences of acute [40,41] and chronic [40,42] resistance exercise. A study that compared the impacts of both modes of exercise determined that the methylome response to aerobic and resistance exercise stimuli is regulated by different molecular pathways [43]. Mode-specific regulation of the methylome is expected considering aerobic and resistance exercise elicit vastly different adaptations [44]; however, both aerobic and resistance exercise result in modifications in methylation for genes associated with inflammatory pathways, indicating that DNA methylation responses are possible mechanisms controlling the impacts of exercise on inflammation.

The supplementation of the diet with fatty acids (FAs), particularly n-3 PUFAs, has been demonstrated to promote an anti-inflammatory phenotype and reduce the concentration of inflammatory cytokines [45,46,47]. While the mechanisms for FA-induced reductions in inflammation remain to be fully elucidated, an epigenetic response following supplementation has been reported [48,49], including for *IL6* DNA methylation [49]. The impact of n-3 PUFAs on exercise-induced inflammation is equivocal, with no consensus existing within the literature. While some studies have identified reductions in exercise-induced inflammation following FA supplementation [50,51], others have reported no change in inflammation [52,53]. The lack of a previous association with exercise-induced inflammation could be a result of using placebos containing other FAs, such as extra virgin olive oil (EVOO), as a comparison; however, these should be investigated independently because of previous reports indicating EVOO supplementation to be sufficient to alter the DNA methylation of genes associated with inflammation [54].

In the current study, we investigated the impacts of acute eccentric resistance exercise on *TNF* and *IL6* DNA methylation and mRNA expression in skeletal muscle and leukocytes in disease-free individuals and examined whether the supplementation of FAs and eccentric resistance training further modified the response. We also investigated the association between skeletal muscle and leukocyte DNA methylation and physiological markers related to exercise performance, inflammation and muscle damage.

## 2. Materials and Methods

### 2.1. Study Participants

The participants (*n* = 8) were healthy, non-smoking males who reported no history of resistance exercise training, metabolic or cardiovascular disease or medication use during the pre-participation health screening. In the six months before the study, the participants had no history of n-3 PUFA, antioxidant or anti-inflammatory supplementation and habitually consumed less than two portions of oily fish per week. The study was approved by the Loughborough University Ethics Human Participants sub-committee (R15-P124).

### 2.2. Study Overview

**Figure 1:**
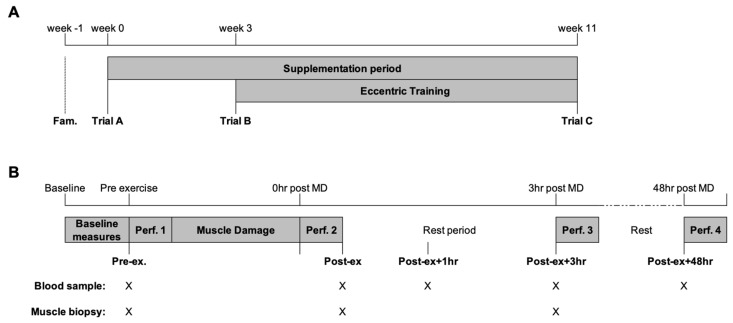
Schematic representation of the (**A**) study and (**B**) trial day. The collection of blood and skeletal muscle tissue is indicated by X. Following the completion of performance test 3, the participants were free to leave the laboratory and returned 30 min before performance test 4 (48 h post-MD). MD, muscle-damaging exercise; Perf., performance test.

A randomised, repeated-measures design with parallel pair-matched groups for isometric and eccentric quadricep strength was used. The study consisted of a familiarisation phase for the study protocols and three experimental trials. The participants self-recorded their dietary intake and physical activity for the 24 h before the initial trial and replicated before each subsequent trial. Between trials, the participants were asked to maintain their habitual diet and report any new instances of medication use. Figure 1 provides a schematic representation of the experimental trials. The first two trials (trial A and trial B) were separated by a three-week double-blind supplementation phase of either n-3 PUFA or EVOO. The participants then completed an eight-week eccentric training program of the knee extensors using the Humac Norm isokinetic dynamometer (CSMI, Stoughton, MS, USA). The participants completed two training sessions per week (minimum of three days between training sessions). The first training session was completed three days following trial B, and the last training session was performed three days before trial C.

On the morning of each trial, the participants reported to the laboratory at the same time of the morning in a fasted and rested state. An intravenous catheter was inserted for the collection of blood samples and the lateral portion of the *vastus lateralis* was prepared under local anaesthesia (1% lidocaine) for the collection of skeletal muscle tissue using the percutaneous needle biopsy technique with suction. Following the collection of baseline samples, the participants completed a performance test followed by a muscle damage protocol. Further skeletal muscle and venous blood samples were collected before performance tests immediately post-exercise (Post-ex) and 3 h post-exercise (Post-ex + 3 h; Figure 1B). The intravenous cannula was removed after completion of the performance test Post-ex + 3 h and the participants were free to leave the laboratory. The participants returned to the laboratory 30 min before the performance test 48 h post-exercise (Post-ex + 48 h) for the collection of venous blood via venepuncture (Figure 1B).

#### 2.2.1. Performance Test

The participants completed a five-minute warm-up on a cycle ergometer (Lode B.V, Groningen Netherlands) at 75 W. The participants then completed countermovement jumps (CMJ) using a Quattro-Jump 9290AD force platform (Kistler, Winterthur, Switzerland). Three CMJs were completed, with one min recovery between efforts; if the peak height was achieved on the final jump, another jump was performed (maximum of five efforts). The participants then performed bilateral maximal voluntary contractions (MVC) of the knee extensors using a Humac Norm isokinetic dynamometer (CSMI, Stoughton, MA, USA). Once positioned on the dynamometer, a warm-up of submaximal contractions (2 × 50%, 1 × 75% and 1 × 90% of perceived MVC; 30 s between efforts) was performed followed by isometric, concentric and eccentric isokinetic MVCs of the knee extensors. For the evaluation of isometric torque, three 3 s isometric contractions of the knee extensors were performed (75° of knee flexion) with a rest period of 30 s between contractions. The maximal concentric and eccentric torque levels were assessed using an angular velocity of 60°/s and a range of motion between 10° and 90° of knee flexion with 30 s rest between contractions. Verbal encouragement and visual feedback were provided. The highest peak torque obtained during the MVCs was used for the analysis.

#### 2.2.2. Eccentric Muscle Damage Protocol

The eccentric muscle damage protocol was performed on the Humac Norm isokinetic dynamometer. The protocol consisted of 20 sets of bilateral maximal voluntary isokinetic eccentric contractions of the knee extensors at an angular velocity of 60°/s using a range of motion between 10 and 90°. Each set consisted of 10 repetitions (reps) and was separated by a one-minute rest period. The participants began with their leg at the start position (10°) and were asked to maximally contract the knee extensors against resistance while the lever arm moved to the finish position (90° knee flexion). Once the lever arm reached 90°, the participants were asked to relax their leg and allow the lever arm to return to the start position (avoiding concentric contraction of the knee extensors). Verbal encouragement and visual feedback (torque output and work done) were provided throughout the muscle damage protocol.

#### 2.2.3. Supplementation

Using a double-blind design, the participants were assigned to either n-3 PUFA (*n* = 4) or EVOO (*n* = 4) supplementation. The groups were counterbalanced for baseline strength measurements. Both the n-3 PUFA (Norwegian Pure-3 AS, Oslo, Norway) and EVOO (Norwegian Pure-3 AS, Oslo, Norway) supplements were provided in capsule form following trial A. The participants were instructed to consume six capsules per day providing 5.1 g of n-3 PUFA (3.0 g of EPA, 1.2 g of DHA and 0.9 g of DPA and other n-3 PUFAs) or 6 g of EVOO per day for the entirety of the study (11 weeks). The dose was chosen based on previous findings showing a similar dose was sufficient to induce changes to the FA profiles of both blood and skeletal muscle [55]. Returned capsules were counted to determine the supplementation compliance.

### 2.3. Collection of Biological Samples

Venous blood samples were collected into K_2_EDTA-coated vacutainers (BD Biosciences, Franklin Lakes, NJ, USA) for analyses of DNA methylation and mRNA expression. Serum samples were isolated from venous blood samples collected in silica-coated vacutainers (BD Biosciences, Franklin Lakes, NJ, USA) for the determination of protein markers of muscle damage and inflammatory cytokines at the Pre-ex, Post-ex, Post-ex + 1 h, Post-ex + 3 h and Post-ex + 48 h timepoints (Figure 1B). Blood cell counts were also performed at each time point using a Yumizen H500 system (Horiba Medical, Kyoto, Japan). Skeletal muscle biopsies were obtained for the determination of DNA methylation and mRNA expression from 6 of the 8 participants (2 participants opted out of biopsies but completed the remaining parts of the study). Following collection, the skeletal muscle tissue was blotted dry and any visible fat or connective tissue was removed, snap-frozen in liquid nitrogen and stored at −80 °C prior to the analysis.

### 2.4. DNA Methylation

Genomic DNA was extracted and bisulfite-converted from both whole blood and skeletal muscle using the EpiTect Fast LyseAll Bisulfite Kit (Qiagen, Hilden, Germany) according to the manufacturer’s instructions. The *TNF* and *IL6* DNA methylation levels were determined using custom PyroMark assays as previously described [39]. Briefly, the bisulfite-converted DNA was amplified using the PyroMark PCR kit (Qiagen, Hilden, Germany) according to the manufacturer’s instructions. The DNA methylation percentage was then determined using a PyroMark Q48 Autoprep system (Qiagen, Hilden, Germany) set in CpG mode using PyroMark Q48 Advanced CpG reagents (Qiagen, Hilden, Germany). A non-CpG cytosine was included in the nucleotide dispensation order to detect incomplete bisulfite conversion.

### 2.5. mRNA Expression

RNA was extracted from whole blood using TRIzol LS (Invitrogen, Waltham, MA, USA) and skeletal muscle using TRI Reagent (Sigma-Aldrich, St. Louis, MO, USA) according to the manufacturer’s instructions. The concentration of RNA isolated from whole blood was 55.36 (±16.60) ng/µL with an A_260_/A_280_ ratio of 1.97 (±0.05), whereas the concentration of RNA isolated from skeletal muscle was 403.34 (±151.99) ng/µL with an A_260_/A_280_ ratio of 2.04 (±0.03). The mRNA expression was then determined as previously described [39]. Briefly, a maximum of 2 µg of RNA was cDNA-converted and the relative mRNA expression for *TNF* and *IL6* was assessed using the 2^−(ΔΔCt)^ method using *GAPDH* as the reference gene [56]. The mean Ct values for *GAPDH* were consistent across all participants and experimental conditions in whole blood (17.31 ± 0.725) and skeletal muscle (12.89 ± 0.475), with low variation rates of 4.18% and 3.68%, respectively.

### 2.6. Protein Markers

The circulating levels of IL-6 and TNF-α were determined using BD™ Cytometric Bead Array Enhanced Sensitivity Flex Sets (BD Bioscience, Franklin Lakes, NJ, USA) on a BD Accuri^TM^ C6 Flow Cytometer (BD Bioscience, Franklin Lakes, NJ, USA) according to the manufacturer’s instructions. The creatine kinase (CK), lactate dehydrogenase (LDH) and myoglobin (Mb) concentrations were determined using ABX Pentra assays (Horiba Medical, Kyoto, Japan) on a Pentra C400 analyser (Horiba Medical, Kyoto, Japan) according to the manufacturer’s instructions. All samples for a participant were performed within a single run to minimise run-to-run variation. Haematocrit and haemoglobin values were used to ascertain the plasma volume changes that were used to adjust the serum concentrations [57].

### 2.7. Statistical Analysis

All statistical analyses were performed using IBM SPSS Statistics software (version 25, IBM, New York, NY, USA). The data were assessed for normality using the Shapiro–Wilk test. All leukocyte DNA methylation analyses were conducted on cell-heterogeneity-adjusted values [58]. DNA methylation differences between tissues at baseline (trial A; Pre-ex) were investigated using *t*-tests. An analysis of mRNA expression was performed on log fold change data. DNA methylation, mRNA expression and physiological markers related to inflammation and muscle damage were analysed using a 3-way between (supplement) × within (trial) × within (time) repeated-measures ANOVA. Where significant effects were observed, the Bonferroni correction was used to control the familywise error rate. Spearman’s Rho correlation analysis was used to assess the relationship between DNA methylation and physiological markers related to exercise performance, inflammation and muscle damage. Moderate (>0.5) correlation coefficients were of interest; however, only large (>0.7) correlation coefficients were deemed statistically significant (*p* < 0.05). All data are presented as means ± 95% CI unless otherwise stated.

## 3. Results

### 3.1. TNF DNA Methylation and mRNA Expression

**Figure 2:**
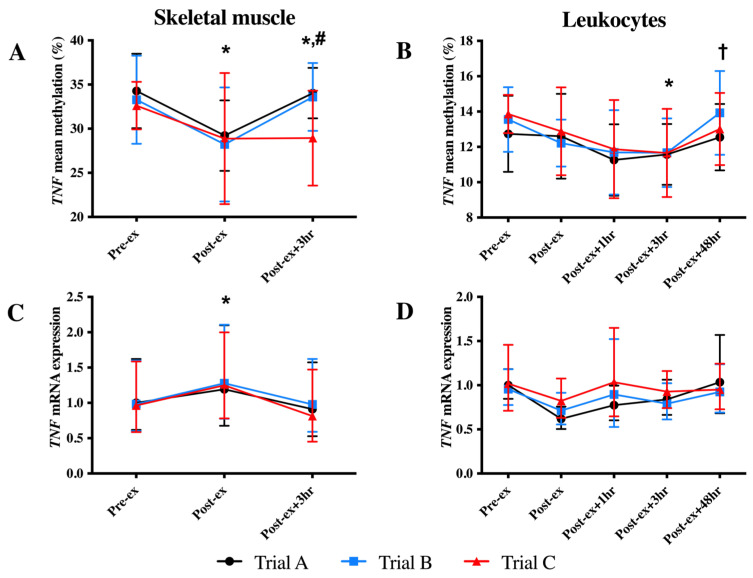
Mean *TNF* DNA methylation (**A**,**B**) and mRNA expression (**C**,**D**) in skeletal muscle (**left-hand column**) and leukocytes (**right-hand column**) in each trial. * Indicates significantly different from Pre-ex; # indicates significantly different from Post-ex; † indicates significantly different from Post-ex + 3 h.

In skeletal muscle, a reduction in the mean DNA methylation of the TNF CpG sites and an increase in TNF mRNA expression were identified at the Post-ex timepoint (*p* < 0.05; Figure 2). The investigation of individual CpG sites identified decreased methylation at Post-ex for two CpG sites (CpG3 and CpG4; *p* < 0.05; Table 1) and non-significant trends for the remaining CpG sites (CpG1: *p* = 0.084; CpG2: *p* = 0.055; Table 1).

In leukocytes, a main time effect was also identified; however, the decrease in *TNF* methylation was identified at Post-ex + 3 h (*p* < 0.05; Figure 2B). The analysis of individual CpG sites identified the association for three CpG sites (CpG2-4: *p* < 0.05; non-significant trend identified for CpG1: *p* = 0.057; Table 1). Despite the change in DNA methylation, exercise was not sufficient to alter the leukocyte *TNF* mRNA expression (*p* > 0.05; Figure 2D).

The supplementation of FAs (trial B) and continued FA supplementation combined with exercise training (trial C) did not alter DNA methylation of *TNF* in either skeletal muscle or leukocytes (Figure 2).

### 3.2. IL6 DNA Methylation and mRNA Expression

**Figure 3:**
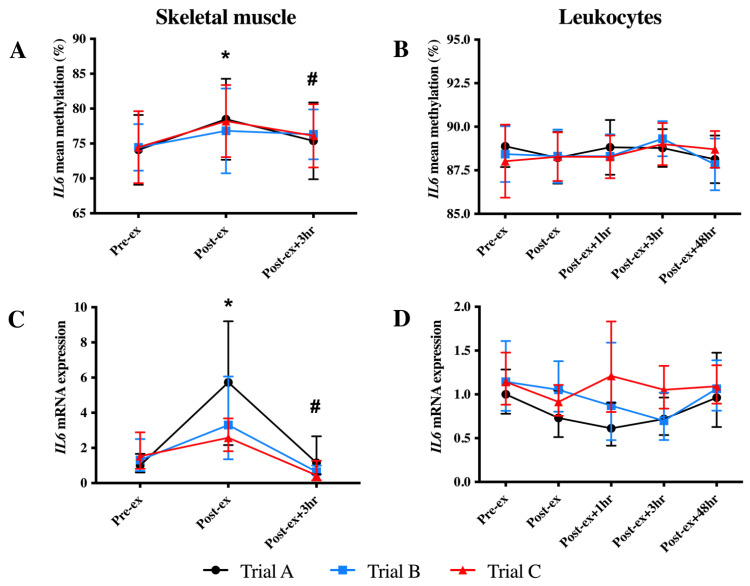
Mean *IL6* DNA methylation (**A**,**B**) and mRNA expression (**C**,**D**) levels in skeletal muscle (left-hand column) and leukocytes (right-hand column) in each trial. Note: * indicates significantly different from Pre-ex; # indicates significantly different from Post-ex.

In skeletal muscle, an increase in the mean *IL6* DNA methylation was identified at Post-ex (*p* < 0.05; Figure 3A). When individual CpG sites were analysed, the increased DNA methylation at Post-ex was significant for each CpG site (*p* < 0.05; Table 2) and decreased to the Pre-ex levels for all CpG sites other than CpG2 and CpG5 (Table 2). Similarly, an immediate increase in skeletal muscle *IL6* mRNA expression at Post-ex was identified; however, mRNA expression returned to Pre-ex expression levels by Post-ex + 3 h (*p* < 0.01; Figure 3C).

There was no significant impact of exercise on the mean leukocyte *IL6* DNA methylation of any CpG sites analysed (*p* = 0.051; Figure 3B). When individual CpG sites were analysed, a main effect of the time was detected for the methylation of two CpG sites (CpG2 and CpG4; *p* < 0.05; Table 2). For CpG2, an increase in methylation was detected at Post-ex + 3 h, which returned to baseline values by Post-ex+ 48 h (*p* < 0.05; Table 2); for CpG4, an immediate decrease in methylation was identified at Post-ex, indicating differential responses between CpG sites (*p* < 0.05; Table 2). The *IL6* mRNA expression in the leukocytes was unaltered by exercise (*p* > 0.05; Figure 3D).

The *IL6* DNA methylation and mRNA expression were unaltered following FA supplementation (trial B) and exercise training (trial C) in skeletal muscle and leukocytes (Figure 3).

### 3.3. Physiological Markers of Inflammation and Muscle Damage

An effect of the time was identified for serum concentrations of IL-6 (*p* = 0.001), CK (*p* = 0.026) and Mb (*p* = 0.002). Compared to Pre-ex, increases in the concentration of these markers were identified at Post-ex and Post-ex + 3 h (*p* < 0.05; Table 3). The circulating concentrations of TNF-α and LDH were unaffected by exercise (Table 3).

Neither FA supplementation or excise training altered the serum concentrations of any of the inflammation or muscle damage markers (*p* > 0.05); however, non-significant trends for the main effect of the trial were detected for CK (*p* = 0.052) and Mb (*p* = 0.087), suggesting a potential reduction in protein concentrations with repeated bouts of exercise (Supplementary Table S1).

### 3.4. Association between DNA Methylation and Physiological Markers

**Figure 4:**
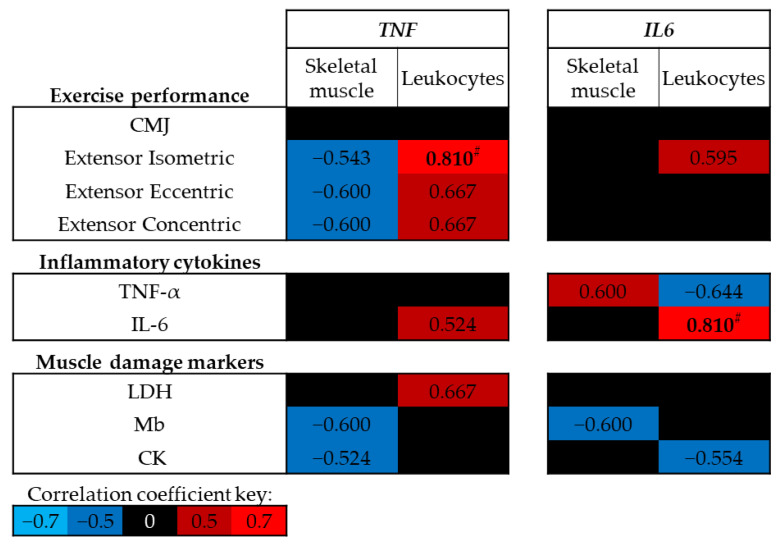
Spearman’s Rho correlation coefficients between baseline (trial A; Pre-ex) DNA methylation and physiological markers related to exercise performance, inflammation and muscle damage. The mean of all CpG sites for each assay has been used to provide an overall view of the region of interest. Blue indicates a negative correlation, red indicates a positive correlation and black indicates correlation coefficients between −0.5 and 0.5. Note # *p* < 0.01. CMJ, countermovement jump; LDH, lactate dehydrogenase; Mb, myoglobin; CK, creatine kinase.

At baseline (trial A, Pre-ex) no significant correlations were identified between exercise performance, circulating levels of inflammatory cytokines or muscle damage markers and skeletal muscle DNA methylation of *TNF* or *IL6* (*p* > 0.05; Figure 4); however, moderate negative correlations were identified between skeletal muscle *TNF* DNA methylation and leg extensor strength. In contrast, positive correlations were identified between leukocyte *TNF* DNA methylation and leg extensor strength, and between leukocyte *IL6* DNA methylation and circulating IL-6 concentrations (*p* < 0.01; Figure 4).

**Figure 5:**
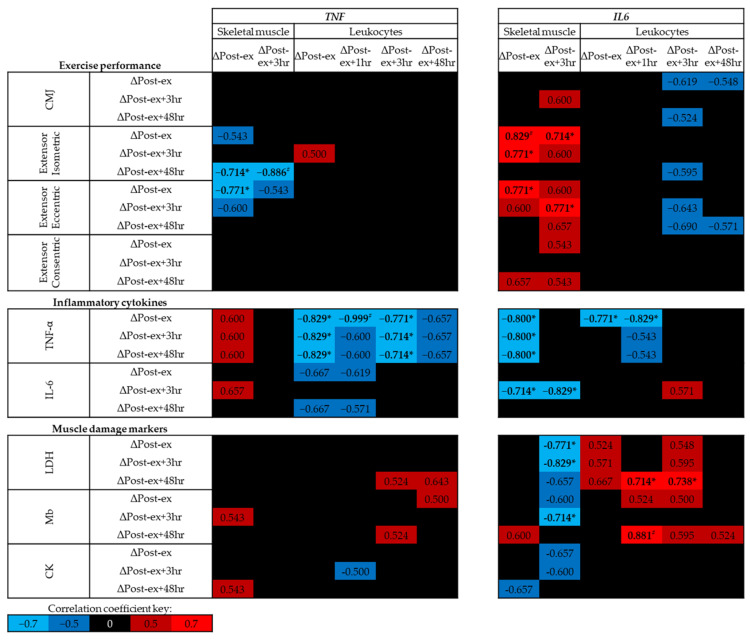
Spearman’s Rho correlation coefficients between changes in DNA methylation at time points post-exercise (post-exercise–pre-exercise) and physiological markers related to exercise performance, inflammation and muscle damage. The mean of all CpG sites assessed for each assay has been used to provide an overall view of the region of interest. Blue indicates a negative correlation, red indicates a positive correlation and black indicates correlation coefficients between −0.5 and 0.5. Note: * *p* < 0.05, # *p* < 0.01. CMJ, countermovement jump; LDH, lactate dehydrogenase; Mb, myoglobin; CK, creatine kinase.

As exercise was sufficient to alter the DNA methylation of *TNF* and *IL6*, the correlation analysis was also performed using the difference between each time point and at Pre-ex for methylation and physiological markers (Figure 5). Negative correlations were identified between the change in skeletal muscle *TNF* DNA methylation and measures of strength following muscle-damaging exercise, whereas positive correlations were identified between the change in *IL6* DNA methylation and measures of exercise performance (*p* < 0.05; Figure 5). No associations were identified between leukocyte *TNF* or *IL6* DNA methylation and exercise performance (*p* > 0.05; Figure 5).

While no association was identified between the changes in skeletal muscle *TNF* DNA methylation and circulating concentrations cytokines or muscle damage markers, strong negative correlations were identified between the changes in skeletal muscle IL6 and circulating concentrations of inflammatory cytokines and muscle damage markers following exercise (*p* < 0.05; Figure 5). The changes in leukocyte *TNF* and *IL6* DNA methylation following exercise were negatively correlated with the expression of *TNF* (*p* < 0.05; Figure 5); however, no correlations were identified with IL-6 concentrations post-exercise. No associations were identified between exercise-induced changes in *TNF* DNA methylation and markers of muscle damage; however, exercise-induced changes in *IL6* DNA methylation were associated with muscle damage markers (Figure 5).

## 4. Discussion

An acute bout of eccentric resistance exercise is sufficient to modulate the DNA methylation and mRNA expression of cytokines (*TNF* and *IL6*) in skeletal muscle and leukocytes from non-resistance-trained males. A bout of eccentric resistance exercise in unaccustomed individuals induced the hypomethylation of *TNF* and hypermethylation of *IL6*. Changes in methylation were detected in both skeletal muscle and leukocytes; however, alterations in mRNA expression were identified only in skeletal muscle. Neither the supplementation of FAs or eight weeks of resistance training further altered the DNA methylation or mRNA expression patterns. Despite similar changes in methylation in skeletal muscle and leukocytes following resistance exercise, differences were identified at baseline (Supplementary Table S2) and negative correlations were determined between tissues. Similarly, contrasting associations were identified between DNA methylation and physiological markers related to exercise performance, inflammation and muscle damage within each tissue, indicating tissue specificity in the methylation response to resistance exercise, which should be considered in future work.

The hypomethylation of the first exon of *TNF* and increased skeletal muscle mRNA expression following acute resistance exercise is a novel finding. Changes in the methylation status in exons are not as well characterised as the methylation of the promoter; however, the methylation of the first exon has been strongly associated with translational silencing [59]. Previous investigations have failed to identify any effect of acute exercise on *TNF* DNA methylation [39,41]; however, the decrease in TNF methylation leading to an increased mRNA expression is supported by a report of decreased *TNF* methylation and increased mRNA expression in a patient population compared to healthy controls [60].

In this study, a tissue-specific response was identified for the time course of TNF hypomethylation, occurring at Post-ex in skeletal muscle, whereas the hypomethylation was delayed until Post-ex + 3 h in leukocytes. In a previous study, we did not observe any change in *TNF* methylation in leukocytes following acute aerobic exercise [39]; however, methylation was only investigated immediately post-exercise in leukocytes. Therefore, in this study, we were unable to identify any changes in methylation at later time points. The only other study to investigate the impact of acute resistance exercise on *TNF* methylation did not document any changes in methylation in skeletal muscle in either trained or untrained individuals [41]. The *TNF* CpG sites analysed in the current study were associated with mRNA and serum levels [39,61,62], whereas the selection of CpG sites in a different region of the *TNF* gene (gene body in the present study vs. promoter region), which may not be functionally relevant for gene expression, may explain the lack of methylation changes in the previous study [41]. Alternatively, the bout of resistance exercise (3 sets of 10 reps at 70% of the 1 repetition maximum on a leg extension machine) used by Bagley et al. [41] may have provided an insufficient stimulus to alter the *TNF* DNA methylation, whereas in the present study, through the inclusion of *TNF* mRNA expression and markers of inflammation and muscle damage, the exercise bout was indicated to be sufficient to induce inflammatory processes.

While there is no previous evidence of altered *TNF* DNA methylation following acute exercise, *TNF* has been reported to be hypermethylated in leukocytes from elderly individuals who maintained or increased their energy expenditure by 500 kcal/week over an eight-year period [63]. These data are supported by evidence of increased *TNF* methylation in the skeletal muscle of resistance-trained compared to sedentary individuals [41]. In the present study, the 8-week resistance training period was insufficient to alter the methylation profile in either skeletal muscle or leukocytes; however, it should be noted that the intervention period in the present study was considerably shorter than in the previous studies (1 year and 8 years). The increased methylation with exercise training suggests the differential regulation of *TNF* by acute resistance exercise and long-term physical activity. The acute decrease in *TNF* methylation and concurrent increase in mRNA expression could be involved in the adaptive response to muscle-damaging exercise via the activation of satellite cells and increased expression of the myogenic differentiation factors MyoD and myogenin [26,27]. Although MyoD and myogenin expression was not determined in the present study, associations between *TNF* methylation and knee extensor force production were identified, suggesting a potential role in the hypertrophic response. However, the increase in *TNF* methylation following long-term physical activity may function to reduce the systemic levels of inflammation associated with disease states and skeletal muscle atrophy [7]. These data suggest a potential epigenetic role for *TNF* in controlling skeletal muscle mass, which is regulated by the stimulus provided by acute and chronic exercise. For the first time, acute eccentric exercise has been demonstrated to be sufficient to alter *IL6* DNA methylation. Immediately following an acute bout of eccentric resistance exercise, *IL6* hypermethylation was identified in skeletal muscle (CpG1-6). Interestingly, the hypermethylation of *IL6* was detected alongside increased *IL6* mRNA expression. The hypermethylation of promoter regions usually results in decreased expression; however, the results are in agreement with a previous report of a positive association between the methylation of a single CpG site (−666) closer to the TSS of the *IL6* promoter and *IL6* mRNA expression [49]. It has been suggested that the increased skeletal muscle production of IL-6 may induce an anti-inflammatory response by increasing the expression of IL-1ra and IL-10 [64] and inhibiting TNF-α production [1,65]. In support of the anti-inflammatory role of muscle-produced IL-6, we identified a negative correlation between skeletal muscle *IL6* methylation and circulating concentrations of TNF-α. The impact of resistance exercise on leukocyte *IL6* methylation is not as clear. In agreement with a previous report [39], acute exercise did not alter the mean methylation of all CpG sites. In the present study, contrasting deviations in methylation were identified at individual CpG sites with decreased methylation at Post-ex at CpG4 and increased methylation at Post-ex + 3 h at CpG2; however, the changes in leukocyte methylation were insufficient to alter the *IL6* mRNA expression. The tissue-specific modulation of *IL6* DNA methylation and mRNA expression suggests that epigenetic mechanisms may be responsible for the increased production of IL-6 in exercising skeletal muscle but not leukocytes [28,66].

The tissue-specific response for methylation following acute eccentric resistance exercise highlights the importance of tissue selection for future studies. Skeletal muscle and leukocytes are both frequently investigated for the impacts of exercise on DNA methylation; however, the methylation responses to exercise have not previously been compared in these tissues. While the DNA sequence is identical across all cells within an individual, the same is not true regarding epigenetic signatures; each tissue and potentially each cell contains a unique methylation profile [67]. The collection of skeletal muscle involves an invasive procedure; therefore, leukocytes are commonly used as a surrogate tissue because of the ease of collection, and as leukocytes circulate throughout the body they interact with various organs and biological systems and are considered a systemic marker of methylation profiles [68]. In the present study, the determination of methylation in both skeletal muscle and leukocytes allows the direct comparison of tissues. For both *TNF* and *IL6,* we identified negative correlations between methylation in skeletal muscle and leukocytes; differences in methylation between skeletal muscle and leukocytes at baseline and in response to acute resistance exercise; and contrasting associations with measures of exercise performance, inflammation and muscle damage. These data suggest that for the CpG sites investigated in the present study, leukocytes should not be used as a surrogate for skeletal muscle DNA methylation investigations; however, these results cannot be generalised to all CpG sites throughout the genome.

Considering the methylation differences between skeletal muscle and leukocytes, an important factor for studies conducting research involving muscle-damaging exercise is the infiltration of leukocytes into skeletal muscle following muscle damage and the potential impact it may have on DNA methylation profiles. The infiltration of leukocytes into skeletal muscle would result in genetic material of leukocytes in skeletal muscle samples, which would impact the determination of DNA methylation. In this study, differences in the methylation profiles of leukocytes and skeletal muscle for each gene were detected at baseline; therefore, if leukocyte infiltration has occurred, it could be the causal factor for the change in DNA methylation following exercise. There is contrasting evidence of the time course of leukocyte infiltration following muscle-damaging exercise; some studies report no infiltration during the initial 3 h post-exercise (the time course of the present study) [69,70,71], while others have reported leukocyte infiltration into skeletal muscle as soon as 30 min post-exercise [72]. Future studies should consider the potential impact of leukocyte infiltration and assess the expression of markers unique to leukocytes to confirm the absence of their contribution to the genetic material used for the skeletal muscle analysis.

While acute resistance exercise was sufficient to alter the DNA methylation patterns in the present study, we did not identify any further impact of exercise training or FA supplementation on DNA methylation. Reductions in systemic levels of inflammation have been reported following exercise training [1,18,19] and FA supplementation [45,46,73,74]. The methylation of a single CpG site—further downstream than the CpG sites in the present study—of the *IL6* gene associated with the n-3 PUFA content in blood [49] and administration of EPA has been reported to dampen the impact of TNF-α on *MyoD* mRNA expression [75]. These data suggest the CpG sites investigated in the present study may regulate the acute local inflammatory response; however, they are not associated with the chronic systemic inflammatory response. Alternatively, the inclusion of young and healthy individuals may have prevented any association due to the lack of a baseline systemic inflammatory response. The repetition of this study in a cohort of older adults with chronic inflammation or within an inflammatory disease population would allow the determination of whether the selected CpG sites are involved only in the acute response or whether the lack of association is due to the selection of young and healthy participants.

The mechanisms responsible for exercise-induced changes to DNA methylation remain to be elucidated. Alterations in the expression and activity of the key enzymes involved in methylation, i.e., DNA methyltransferases (DNMT), are mechanisms that have been previously demonstrated [39,76,77]. In agreement with previous reports, we identified altered *DNMT3a* and *DNMT3b* mRNA expression following exercise (Supplementary Figure S1); however, the expression profile of these enzymes was not correlated with either *TNF* or *IL6* methylation. As DNMT3a and 3b are responsible for de novo methylation, the gene-specific approach adopted in the present study may explain the lack of association with *TNF* and *IL6* DNA methylation. While it is unknown how exercise influences DNMT expression, a potential mechanism is via miRNA expression. Exercise alters the expression of various miRNAs, including *miR-29-130* and *-148* [78], which are also associated with the expression of DNMTs [79,80,81,82]. Future work should include measures of DNMT enzyme activity and extend the analysis to also include an assessment of the TET enzymes responsible for DNA demethylation to investigate potential mechanisms of exercise-induced DNA methylation.

## 5. Conclusions

Acute eccentric resistance exercise was sufficient to alter the DNA methylation of *IL6* and *TNF* in skeletal muscle and leukocytes; however, resistance training and FA supplementation did not alter the methylation profiles further. Baseline differences and a tissue-specific response following exercise were determined between skeletal muscle and leukocytes. The tissue specificity was further demonstrated by contrasting associations with markers of exercise performance, inflammation and muscle damage within skeletal muscle tissue and leukocytes. The tissue-specific response between skeletal muscle and leukocytes is an important finding because leukocyte methylation is commonly used as a surrogate for other tissues. The lack of alteration of DNA methylation because of exercise training suggests that these methylation changes occur independently of the training status; however, this may be due to the selection of a young cohort of healthy males with a lack of chronic inflammation. Future work should investigate the epigenetic impact of chronic exercise and nutritional interventions in individuals suffering from inflammatory diseases.

## Figures and Tables

**Table 1:** DNA methylation of *TNF* CpG sites. Data presented as the mean of all trials ± standard deviation.

CpG site	Tissue	Pre-Ex	Post-ex	Post-ex + 1 h	Post-ex + 3 h	Post-ex + 48 h	*p*
*TNF* CpG1: +197	Skeletal muscle	29.63 ± 3.04	26.21 ± 3.98	N.D.	29.17 ± 3.32	N.D.	0.084
	Leukocytes	13.77 ± 1.55	13.01 ± 2.05	12.06 ± 1.81	12.17 ± 2.03	13.45 ± 1.97	0.057
*TNF* CpG2: +202	Skeletal muscle	24.32 ± 2.70	20.82 ± 3.40	N.D.	23.35 ± 2.83	N.D.	0.055
	Leukocytes	11.2 ± 1.61 ^a^	10.86 ± 1.66 ^ab^	10.04 ± 1.96 ^ab^	10.01 ± 1.75 ^b^	11.09 ± 1.57 ^ab^	0.048
*TNF* CpG3: +214	Skeletal muscle	29.09 ± 2.59 ^a^	25.37 ± 4.03 ^b^	N.D.	27.87 ± 3.09 ^ab^	N.D.	0.044
	Leukocytes	12.97 ± 1.82 ^a^	12.43 ± 2.00 ^ab^	11.67 ± 2.23 ^ab^	11.58 ± 1.93 ^b^	12.7 ± 1.99 ^ab^	0.020
*TNF* CpG4: +222	Skeletal muscle	50.53 ± 3.95 ^a^	42.7 ± 6.51 ^b^	N.D.	48.37 ± 5.09 ^ab^	N.D.	0.012
	Leukocytes	15.60 ± 1.76 ^a^	13.97 ± 2.05 ^ab^	12.6 ± 2.00^ab^	12.78 ± 1.66 ^b^	15.41 ± 2.23 ^a^	0.001

Values not sharing a letter (^a,b^) are significantly different for simple interactions of time (*p* < 0.05) after Bonferroni correction for multiple tests. N.D., not determined.

**Table 2:** DNA methylation of *IL6*. Data presented as the mean of all trials ± standard deviation.

CpG Site	Tissue	Pre-Ex	Post-Ex	Post-Ex + 1 h	Post-Ex + 3 h	Post-Ex + 48 h	*p*
*IL6* CpG1: -1099	Skeletal muscle	73.06 ± 4.33 ^a^	77.96 ± 5.68 ^b^	N.D.	76.06 ± 3.48 ^ab^	N.D.	**0.009**
	Leukocytes	90.81 ± 1.57	90.53 ± 1.05	91.12 ± 1.35	91.6 ± 1.22	90.57 ± 1.54	0.308
*IL6* CpG2: -1096	Skeletal muscle	77.40 ± 3.14 ^a^	81.01 ± 3.87 ^b^	N.D.	79.04 ± 3.08 ^c^	N.D.	**0.001**
	Leukocytes	90.56 ± 1.21 ^a^	91.14 ± 1.16 ^ab^	91.25 ± 1.09 ^ab^	91.76 ± 0.81 ^b^	90.67 ± 0.76 ^a^	**0.029**
*IL6* CpG3: -1094	Skeletal muscle	82.61 ± 3.28 ^a^	84.87 ± 3.28 ^b^	N.D.	83.44 ± 2.86 ^a^	N.D.	**0.025**
	Leukocytes	90.96 ± 2.17	91.1 ± 2.18	91.06 ± 2.54	91.79 ± 1.86	90.76 ± 1.99	0.098
*IL6* CpG4: -1069	Skeletal muscle	66.13 ± 3.74 ^a^	70.78 ± 5.24 ^b^	N.D.	67.31 ± 3.84 ^a^	N.D.	**0.002**
	Leukocytes	88.88 ± 1.66 ^a^	87.52 ± 1.90 ^b^	88.28 ± 1.47 ^ab^	89.23 ± 1.23 ^ab^	88.36 ± 1.34 ^ab^	**0.016**
*IL6* CpG5: -1061	Skeletal muscle	72.41 ± 2.87 ^a^	74.88 ± 3.38 ^b^	N.D.	73.83 ± 3.42 ^c^	N.D.	**0.001**
	Leukocytes	81.52 ± 2.98	80.91 ± 2.51	81.54 ± 1.98	81.91 ± 2.27	81.27 ± 2.82	0.166
*IL6* CpG6: -1057	Skeletal muscle	74.46 ± 3.74 ^a^	77.55 ± 4.23 ^b^	N.D.	75.93 ± 3.86 ^a^	N.D.	**0.001**
	Leukocytes	87.94 ± 2.17	88.43 ± 1.59	87.64 ± 1.43	87.91 ± 1.36	87.71 ± 2.10	0.595

Values not sharing a letter (^a,b,c^) are significantly different for simple interactions of time (*p* < 0.05) after Bonferroni correction for multiple tests. N.D., not determined.

**Table 3:** Serum concentrations of protein markers associated with inflammation and muscle damage.

Marker	Pre-Ex	Post-Ex	Post-Ex + 3 h	Post-Ex + 48 h	*p*
TNF-α (pg/mL)	0.21 ± 0.17	0.19 ± 0.09	0.27 ± 0.18	0.25 ± 0.15	0.478
IL-6 (pg/mL)	0.46 ± 0.17 ^a^	3.77 ± 2.28 ^b^	2.90 ± 1.35 ^b^	1.17 ± 1.22 ^ab^	**0.001**
LDH (U/L)	222.62 ± 70.60	240.62 ± 67.16	272.67 ± 68.37	264.78 ± 81.85	0.462
Mb (µg/L)	45.38 ± 24.62 ^a^	284.55 ± 167.62 ^b^	328.68 ± 199.12 ^b^	143.47 ± 206.99 ^ab^	**0.002**
CK (U/L)	149.58 ± 26.44 ^a^	275.45 ± 78.98 ^b^	479.73 ± 225.80 ^b^	586.50 ± 332.94 ^ab^	**0.026**

Data presented as the mean of all trials ± standard deviations. Values not sharing a letter (^a,b^) are significantly different for simple interactions of time (*p* < 0.05) after Bonferroni correction for multiple testing. LDH, lactate dehydrogenase; Mb, myoglobin; CK, creatine kinase.

## Data Availability

The study datasets and protocols of the current manuscript are available from the corresponding author upon request.

## Supplementary Materials

The following supporting information can be downloaded at: https://www.mdpi.com/article/10.3390/genes14020478/s1. Figure S1: Effect of exercise on the mRNA expression of (A,B) DNMT1, (C,D) DNMT3a and (E,F) DNMT3b in skeletal muscle (left-hand column) and leukocytes (right-hand column). * Indicates significantly different from Pre-ex; # indicates significantly different from Post-ex. Table S1. Serum concentrations of protein markers associated with inflammation and muscle damage. Data presented as the mean of each trial ± standard deviations. LDH, lactate dehydrogenase; Mb, myoglobin; CK, creatine kinase. Table S2. Methylation of skeletal muscle and leukocytes at baseline (trial A, Pre-ex). Note: *p* < 0.05 indicates a significant difference between tissues. Data presented as means ± SD.
