# Supplementary material for: Impacts of Eccentric Resistance Exercise on DNA Methylation of Candidate Genes for Inflammatory Cytokines in Skeletal Muscle and Leukocytes of Healthy Males
Source: Genes (Basel). 2023 Feb 13;14(2):478. doi: 10.3390/genes14020478 (PMC9957508; doi:10.3390/genes14020478)
Supplement: Supplementary file 1 [file genes-14-00478-s001.zip › genes-2185665-supplementary.pdf]

Supplementary Table S1. Serum concentrations of protein markers associated with inflammation and muscle damage. Data presented as the mean of each trial ± standard deviations. LDH; Lactate dehydrogenase, Mb; Myoglobin, CK; Creatine Kinase.

Marker	Trial	Pre-ex	Post-ex	Post-ex+3hr	Post-ex+48hr
TNF-α (pg/mL)	A	0.26 ± 0.25	0.23 ± 0.19	0.29 ± 0.27	0.25 ± 0.22
	B	0.26 ± 0.29	0.16 ± 0.13	0.21 ± 0.06	0.21 ± 0.23
	C	0.12 ± 0.06	0.2 ± 0.11	0.27 ± 0.32	0.30 ± 0.27
IL-6 (pg/mL)	A	0.36 ± 0.27	3.73 ± 1.8	3.4 ± 2.73	2.16 ± 2.86
	B	0.5 ± 0.39	2.66 ± 1.88	2.68 ± 1.15	0.66 ± 0.83
	C	0.52 ± 0.19	4.91 ± 4.64	2.64 ± 1.3	0.7 ± 0.68
LDH (U/L)	A	214.74 ± 71.44	238.16 ± 66.88	293.28 ± 84.59	291.44 ± 114.51
	B	226.48 ± 92.25	240.49 ± 85.9	259.74 ± 77.61	254.97 ± 84.96
	C	226.64 ± 65.12	243.23 ± 63.16	265 ± 65.04	247.95 ± 67.79
Mb (µg/L)	A	42.49 ± 22.35	438.18 ± 314.56	549.96 ± 399.67	364.14 ± 611.31
	B	35.31 ± 11.26	201.43 ± 121.66	210.97 ± 134.8	34.23 ± 11.81
	C	58.35 ± 72.42	214.03 ± 126.92	225.1 ± 114.79	32.02 ± 9.15
CK (U/L)	A	140.88 ± 26.47	280.69 ± 115.18	610.14 ± 424.22	889.98 ± 714.5
	B	163.38 ± 65.72	264.35 ± 111.26	397.45 ± 200.04	429.48 ± 220.21
	C	144.5 ± 49	281.31 ± 118.28	431.61 ± 218.18	440.04 ± 234.83

Supplementary Table S2. Methylation of skeletal muscle and leukocytes at baseline (Trial A, Pre-ex). p<0.05 indicates a significant difference between tissues. Data presented as mean ± SD.

CpG Site	Tissue	Methylation (%)	p
TNF			
CpG1	Skeletal Muscle	30.82 ± 5.11	**<0.001**
	Leukocytes	13.17 ± 2.70	
CpG2	Skeletal Muscle	25.03 ± 3.88	**<0.001**
	Leukocytes	10.53 ± 2.5	
CpG3	Skeletal Muscle	30.41 ± 3.17	**<0.001**
	Leukocytes	12.62 ± 3.42	
CpG4	Skeletal Muscle	50.87 ± 5.64	**<0.001**
	Leukocytes	14.65 ± 3.31	
CpG Mean	Skeletal Muscle	34.28 ± 4.38	**<0.001**
	Leukocytes	12.74 ± 2.58	
IL6			
CpG1	Skeletal Muscle	72.41 ± 6.93	**0.002**
	Leukocytes	91.10 ± 1.92	
CpG2	Skeletal Muscle	76.87 ± 4.46	**<0.001**

	Leukocytes	91.12 ± 1.19	
CpG3	Skeletal Muscle	82.94 ± 3.97	**0.008**
	Leukocytes	91.17 ± 3.09	
CpG4	Skeletal Muscle	66.08 ± 5.03	**<0.001**
	Leukocytes	88.89 ± 1.95	
CpG5	Skeletal Muscle	72.23 ± 4.03	**0.007**
	Leukocytes	82.01 ± 3.58	
CpG6	Skeletal Muscle	74.09 ± 5.38	**<0.001**
	Leukocytes	88.99 ± 2.3	
CpG Mean	Skeletal Muscle	74.10 ± 4.14	**<0.001**
	Leukocytes	88.87 ± 1.42	

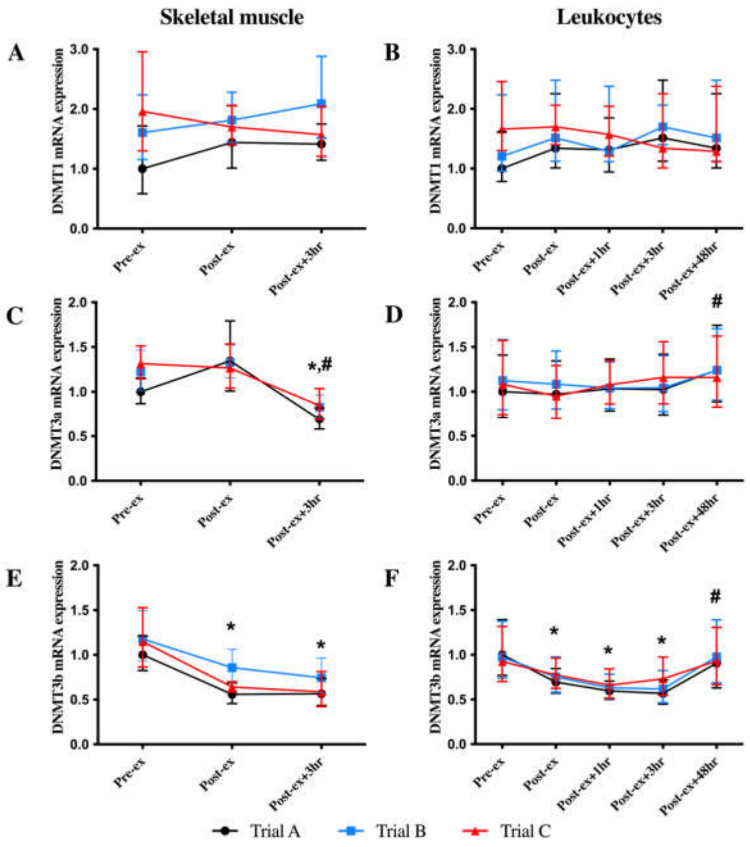

Supplementary Figure S1. Effect of exercise on the mRNA expression of (A&B) DNMT1, (C&D) DNMT3a and (E&F) DNMT3b in skeletal muscle (left-hand column) and leukocytes (right-hand column). *Indicates significantly different from Pre-ex; # indicates significantly different from Post-ex.
